# Unveiling soil thermal behavior under ultra-high voltage power cable operations

**DOI:** 10.1038/s41598-025-91831-1

**Published:** 2025-03-01

**Authors:** Shahbaz Ahmad, Zarghaam Haider Rizvi, Frank Wuttke

**Affiliations:** 1 Geomechanics and Geotechnics, University of Kiel, Kiel, Germany; 2 Civil and Environmental Engineering, University of Waterloo, Waterloo, ON Canada; 3 Kiewit Inc., Denver, USA

**Keywords:** Underground power cables, Energy geotechnics, Cyclic thermal loading, Natural convection, Thermal charging, Green energy systems, Energy infrastructure, Power distribution, Civil engineering, Electrical and electronic engineering

## Abstract

The optimal operation of high-voltage underground power cables is crucial for powering our communities, and it hinges on the intricate dynamics of insulation temperature around the conductor, primarily influenced by joule heating. This temperature responsiveness is further molded by seasonal and diurnal fluctuations in power demand, as well as the moisture content in the surrounding soil. Past research concentrated on theoretical analyses and experiments under dry conditions, but our study expands this scope. Through extensive laboratory tests exploring static and cyclic thermal loads in both dry and saturated sand environments, we uncovered valuable insights. Cyclic thermal loads in dry sand demonstrated a significant thermal charging effect, especially with shorter relaxation times. In static thermal loading, utilizing saturated sand enhanced heat dissipation due to higher thermal conductivity. However, it also revealed a noteworthy observation: a robust convection cell formed after three days of continuous heating, presenting challenges for cables under crop fields despite facilitating efficient cooling. Highlighting the importance of high-voltage power cable infrastructure, our study delves into the critical intersection between infrastructure and the underground soil. Understanding these interactions becomes imperative for the sustainable development of clean energy initiatives. As the world transitions to cleaner energy practices, optimizing the performance of underground power cable systems becomes pivotal in realizing their full potential and aligning with broader clean energy goals. This research contributes essential knowledge to enhance the safety, efficiency, and sustainability of high-voltage underground power cable systems in support of a cleaner and more sustainable energy future.

## Introduction

**Figure 1:**
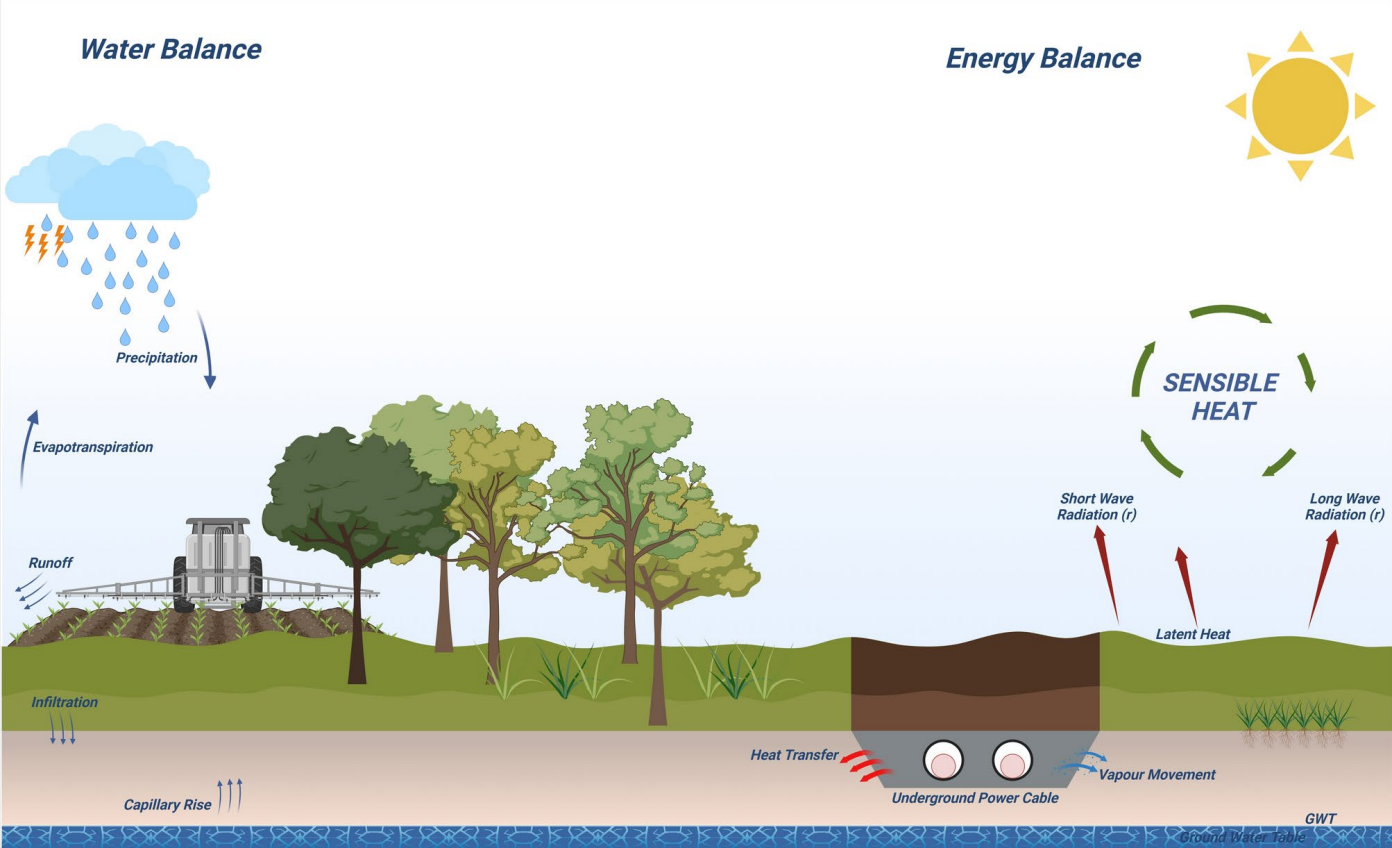
Heat and mass transfer dynamics between underground power cables and the surrounding soil environment. (This figure was created in BioRender.AHMAD, S. (2025) https://BioRender.com/j37i818).

Power transmission and distribution are pivotal to ensuring energy security and fostering industrial growth. Long-distance power transmission is typically achieved through high-voltage transmission lines. However, these systems face various environmental challenges, such as high winds, snow and ice storms, earthquakes, and incidents of metal wire theft^1,2^. Another significant concern is electromagnetic pollution associated with overhead lines, often manifested as hissing or humming noises^3^. These challenges have prompted a shift toward underground power cable systems (UGPS), which offer a more reliable and environmentally friendly alternative for energy transmission. The integration of decentralized green energy systems, including wind, solar, and tidal power generation from strategically favorable sites, has further highlighted the importance of UGPS. Transmitting power to industrial consumption centers via high-voltage underground cables mitigates the environmental and spatial constraints posed by overhead lines^4,5^. Consequently, understanding the thermal and mechanical behavior of UGPS has become a critical research focus over the past few decades. While underground power cable systems are integral to modern infrastructure, their thermal management presents unique challenges. Efficient heat dissipation depends on several factors, including soil thermal properties, moisture content, and environmental conditions. Addressing these challenges is essential to ensure the reliability and longevity of these systems. This study focuses on understanding these complexities and providing actionable insights through experimental and modeling approaches. As shown in Fig. 1, the interaction between heat generation within the cable and the surrounding soil environment is governed by complex heat and mass transfer dynamics, including Joule heating, dielectric losses, and moisture migration. These processes significantly influence the cable’s thermal performance, making their investigation essential for optimizing underground power cable systems. A primary limitation in UGPS operation is the melting temperature of cross-linkable polyethylene (XLPE), which surrounds the conductor. Joule heating, caused by resistance in electric current flow, raises the temperature of XLPE, potentially compromising its performance^6–8^. Effective heat dissipation into the surrounding soil is therefore crucial for enhancing the current-carrying capacity (ampacity) of UGPS. Various methods, including optimized cable arrangements^9–13^, and the use of trench backfill materials^14–17^, have been explored to address this challenge. Most theoretical and experimental investigations, as well as industry standards like the International Electrotechnical Commission (IEC) guidelines, assume a static equilibrium between the cable and the surrounding soil. This results in the formation of an isothermal boundary around the cables. Additionally, Joule heating often leads to moisture migration from the soil near the cable. The most unfavorable scenario occurs under dry soil conditions, where reduced thermal conductivity increases the risk of “hot spots.” While these static assumptions provide a useful baseline, they are often unrealistic for high-voltage UGPS (above 345 kV), particularly in dynamic conditions associated with green energy transmission. Recent numerical studies^18^ and theoretical investigations^19^ have begun to incorporate dynamic influences, such as the effects of daily load cycles on UGPS. These studies suggest that dynamic thermal analysis could enable cables to carry more power. Additionally, numerical modeling techniques, such as the Lattice Element Method (LEM), have been employed to simulate stress localization and fracture behavior in cemented geomaterials under dynamic loading conditions^20^. However, there is a lack of experimental research investigating cyclic thermal loading and a comparative analysis of dry and saturated soil conditions. Experimental programs addressing temperature and moisture field distributions around UGPS are typically conducted at field or laboratory scales^21^. Field-scale experiments provide valuable real-world data but are resource-intensive and challenging due to uncontrollable boundary conditions and soil heterogeneity^22,23^. For instance, Trinks^24^ conducted field studies on 110 *kV* cables, while Ainhirn^25^ analyzed the sensitivity of environmental and material parameters affecting cable temperature. Although insightful, such studies often produce limited datasets due to the complexity of field conditions. Medium-scale laboratory experiments have gained popularity for their practicality and cost-effectiveness^26^. These setups often use rectangular boxes with cylindrical heat sources to simulate UGPS behavior under controlled conditions^27,28^. For example, Vollaro et al.^29^ examined the effects of non-homogeneous soil and backfill materials on thermal fields, while Salata et al.^16,30^ proposed modifications to IEC methods based on experimental data. However, medium-scale studies are limited by boundary effects and heat accumulation in the surrounding soil. To overcome these limitations, large-scale testing boxes equipped with single or multiple heaters have been developed^4,31^. These setups allow for better control of boundary effects and provide deeper insights into heat dissipation mechanisms in UGPS. Our study employs such a large-scale experimental approach to investigate the thermal behavior of UGPS under dry and saturated soil conditions. These two conditions represent the extreme scenarios critical for cable design, as they define the range of possible thermal resistivities. This approach aligns with standard practices in thermal analysis, where evaluating soil properties under moisture extremes is essential for accurate cable ampacity calculations^32,33^. In this work, we introduce a dynamic perspective to traditional static equilibrium assumptions. Our experimental results, presented through thermal contour plots and graphs, reveal significant findings such as dynamic equilibrium and convection cell formation in saturated soils. These insights serve as benchmarks for numerical modeling and provide a foundation for optimizing UGPS performance under real-world conditions. Additionally, this study lays the groundwork for future investigations into the behavior of unsaturated soils, which are more representative of natural field conditions. Understanding the thermal and mechanical behavior of UGPS is an interdisciplinary challenge requiring insights from geotechnical, thermal, and electrical engineering. This manuscript examines these complexities, offering strategies to improve the efficiency and sustainability of underground power cable systems.

## Theory of coupled heat and mass transport in sand

The theoretical and numerical examination of heat and mass transfer around the Underground Power Cable System (UGPS) relies on the foundational assumption of static equilibrium and isotherm. However, the intricacies of heat and mass transfer in porous media, such as soil, involve complex interactions among gas, heat, liquid water, and vapor flow. This study delves into a comprehensive analysis of heat and mass transfer in sand based on the thermodynamics of irreversible processes^34^. While some alignment between theory and experiment exists, it is only partial and necessitates numerous assumptions. Another approach involves a ’mechanistic’ explanation of the process, commonly applied to various porous media due to the simplicity in computing diffusion parameters^35^. However, this theory has limitations, including a hysteresis relation in moisture potential, a lack of consideration for moisture content, and the requirement of a homogeneous and isotropic macroscopic sense in the porous medium. The model excludes the deformation of the solid matrix, as well as phase change phenomena like boiling, freezing, and thawing. Additionally, it doesn’t account for the Knudsen effect in the gas phase and surface phenomena at the matrix-liquid interface. Several simplifications are incorporated into the formulation of the governing coupled partial differential equations. For fluids within the porous media, the assumption is that solutes are absent, meaning the liquid phase is pure water, and movement is driven by viscous flow influenced by capillary and adsorptive forces. Similarly, vapor movement occurs through diffusion in gas-filled pores, with air as an inert gas, and free convection in the gas phase is neglected. Regarding heat transfer, the model assumes negligible radiation effects, maintaining the assumption of local thermodynamic equilibrium among the liquid in contact with its vapor. It also assumes uniform and constant total pressure, while disregarding the temperature dependence of physical constants^36^. With these identified limitations and assumptions, the mass balance in the porous media is described as follows:

The liquid flux density

1 $$\begin{aligned} q_{l}=-\rho _{l}(D_{\theta l}\nabla \theta _{l}+D_{Tl}\nabla T+{K}\textbf{k}) \end{aligned}$$

where, $D_{T l}$ and $D_{\theta l}$ are the thermal liquid and isothermal diffusivities respectively

2 $$\begin{aligned} D_{\theta l} = K \partial \psi /\partial \theta _{l}, \; D_{T l} = K \partial \psi /\partial T \end{aligned}$$

The total water vapour flux is the summation of the isothermal flux component and the temperature-driven flux component

3 $$\begin{aligned} & {\textbf{q}}_{v} = -\rho _{l}(D_{\theta v} \nabla \theta _{l} + D_{Tv} \nabla T) \end{aligned}$$

4 $$\begin{aligned} & D\theta _{v} = f(a)D\frac{P}{P-p_{v}} \frac{Mg}{RT} \frac{\rho _{v}}{\rho _{l}} \frac{\partial \psi }{\partial \theta _{l}} \end{aligned}$$

5 $$\begin{aligned} & D_{Tv} = f(a)D\frac{P}{P-p_{v}}\frac{\rho _{v}}{\rho _{l}} \frac{\zeta }{p_{vs}}\frac{dp_{vs}}{dT} \end{aligned}$$

The partial pressure of water vapour is related to the soil water potential through the fractional relative humidity as

6 $$\begin{aligned} & {\textbf{p}}_{v}= hp_{vs} = p_{vs}exp(Mg\psi /RT) \end{aligned}$$

7 $$\begin{aligned} & f(a) = {\left\{ \begin{array}{ll} a + \theta _{l} = S, & \text {for } \theta _{l} \le \theta _{lk}, \\ a + a \frac{S-a}{S-\theta _{lk}}, & \text {for } \theta _{l} > \theta _{lk}. \end{array}\right. } \end{aligned}$$

8 $$\begin{aligned} & \zeta = (\nabla T)_{a}/\nabla T \end{aligned}$$

The total moisture flux is the combination of liquid and vapour fluxes

9 $$\begin{aligned} \varvec{{\textbf{q}}_{m} = {\textbf{q}}_{l} +{\textbf{q}}_{v} }= -\rho _{l}(D_{\theta }\nabla \theta _{l} + D_{T} \nabla T+\varvec{ K}{\textbf{k}}) \end{aligned}$$

Similarly, the total energy transport in the porous media is the summation of sensible, convective and latent heat fluxes

10 $$\begin{aligned} \varvec{{\textbf{q}}_{h}}= -\lambda \nabla T+c_{1} (T-T_{0}){\textbf{q}}_{m}-L\rho _{1}D_{\theta v}\nabla \theta _{l} \end{aligned}$$

The transport equations described above for liquid water, heat and water vapour are combined to obtain a coupled system of two partial differential equations, with temperature *T* and volumetric moisture content $\theta _{l}$,

11 $$\begin{aligned} & \left\{ l+ \frac{(S-\theta _{l})\rho _{v}}{\rho _{l}} \frac{Mg}{RT} \frac{\partial \psi _{l}}{\partial \theta }\ -\frac{\rho _{v}}{\rho _{l}} \right\} \frac{\partial \theta _{l}}{\partial _{t}}+\frac{(S-\theta _{1})h}{\rho _{l}} \frac{d\rho _{vs}}{dT}\frac{\partial T}{\partial t} \nonumber \\ & =\nabla (D_{\theta }\nabla \theta _{l})+\nabla (D_{T}\nabla T)+\partial {\textbf{K}}/\partial z \end{aligned}$$

12 $$\begin{aligned} & \left\{ \frac{L(S-\theta _{l})\rho _{v}Mg}{RT} \frac{\partial \psi }{\partial \theta _{l} }-L\rho _{v}\right\} \frac{\partial \theta _{l}}{\partial t} +\left\{ C+L(S-\theta _{l})h\frac{d\rho _{vs}}{dT} \right\} \frac{\partial T}{\partial t}= \nabla (\lambda \nabla T)+ L\rho _{l} \nabla (D_{\theta v}\nabla \theta _{l})\nonumber \\ & \quad -c_{l}(q_{l}\nabla T)- c_{pv}(q_{v}\nabla T) \end{aligned}$$

The preceding equations are resolved using the finite element method^37^ and finite difference method^38^ under atmospheric boundary conditions for the Underground Power Cable System (UGPS) to determine temperature and moisture fields. However, the equations formulated under the “mechanistic” approach exhibit significant errors at both low and high moisture values, especially when saturation levels change rapidly. Additionally, these equations do not account for the thermal buoyancy effect, making it challenging to predict fully saturated density-driven flow accurately. Therefore, to achieve precise estimations for the intricate problem of the temperature field around a heater, experimental studies are deemed ideal and are undertaken in this context.

## Material and method

**Figure 2:**
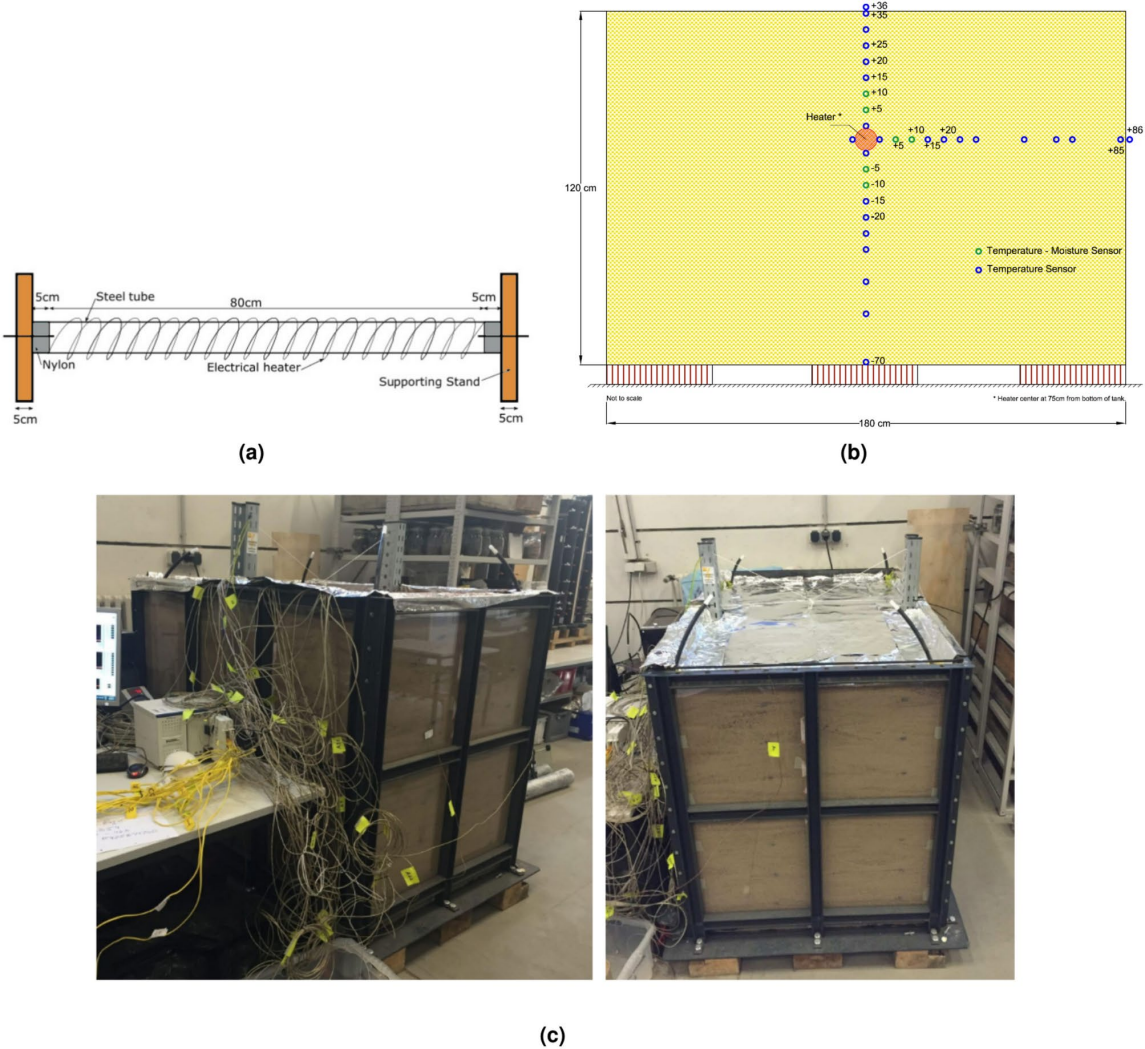
Illustration of the experimental setup: (**a**) heating rod with holding staffs, (**b**) thermocouple placement in vertical and horizontal planes, and (**c**) experimental setup used in the study.

The construction of the large-scale testing box incorporates supporting staffs (vertical beams) for the heater, with the plate strategically mounted on wooden beams to ensure no direct contact with the surface. The sides of the box are reinforced with a robust C-section, capable of withstanding the load exerted by the filled and compacted sand. A cylindrical heater is securely fastened using a bolt mechanism, with the ends insulated using a nylon block to prevent heat loss (refer to Fig. 2a). Transparent acrylic glass forms the side boundaries, facilitating observation of the experiment’s progress and acting as an effective insulator. The sand utilized for the test is sourced from a site near Kiel, Germany, with its chemical and thermo-physical properties detailed in Tables 1 and 2. While a brief description of the experimental assembly is provided here, a more comprehensive overview can be found in the work by Ahmad et al.^4^.

### Sand

X-ray fluorescence (XRF) analysis of the sand confirms that its primary component is silica, with trace amounts of other chemical elements (Table 1). The sand predominantly consists of quartz, the crystalline form of silica (SiO_2_), which accounts for 99.28% of its composition. The thermophysical properties of the sand are summarized in Table 2. Geotechnical laboratory tests indicate that the sand is classified as uniform sand, with a porosity of 0.36. The measured thermal conductivity values are 0.365 W/m K for dry sand and 2.54 W/m K for fully saturated sand.

**Table 1:** X-ray fluorescence (XRF) results for the sand.

Weight percent oxides of the soil sample						
Compound	$SiO_{2}$	$Al_{2}O_{3}$	$Fe_{2}O_{3}$	*CaO*	*MgO*	$K_{2}O$
Percentage	99.27819	0.193343	0.078948	0.04028	0.098282	0.019334

**Table 2:** Thermo-physical properties of sand.

Properties	Values
Gravel, > 2 mm (wt.%)	4.78
Sand, 0.063-2 mm (wt.%)	95.16
Silt and clay, < 0.063 mm (wt.%)	0.06
Porosity *n* (–)	0.36
Solids specific gravity $G_{s}$ (–)	2.66
Dry Density $\rho _{d}$ (kg m$^{-3}$)	1720
Grain diameter at $10\%$ passing $D_{10}$ (mm)	0.27
Grain diameter at $50\%$ passing $D_{50}$ (mm)	0.60
Coefficient of uniformity $C_{u}$ (–)	2.63
Coefficient of curvature $C_{c}$ (–)	0.88
Dry effective thermal conductivity $\lambda _{dry}$ (W m$^{-1}$ K$^{-1})^\textrm{a}$	0.365
Saturated eff. thermal conductivity $\lambda _{sat}$ (W m$^{-1}$ K$^{-1})^\textrm{a}$	2.54
Dry effective specific heat capacity $c_{dry}$ (M J m$^{-3}$ K$^{-1})^\textrm{a}$	1.39
Saturated eff. specific heat capacity $c_{sat}$ (M J m$^{-3}$ K$^{-1})^\textrm{a}$	2.453
Unified soil classification system (USCS)	$SP^\textrm{b}$

$^\textrm{a}$Effective parameters obtained using Decagon *KD*2 Pro transient thermal needle probes. $^\textrm{b}$Poorly graded sand

### Experimental setup

The rectangular box for the test is 1800 mm long, 1000 mm wide and 1200 mm high (see Fig. 2b, c). The experimental setup is the same as in Ahmad et al.^4^ with minor changes in the data acquisition setup to study relaxation time and to deal with saturated conditions. The fabricated container had clear acrylic glass faces on all four sides for precise observation which were bevelled on the edges and top to ensure safety for the working personnel. The container was secluded directly from the ground using solid wooden planks and rubber sheets to remove any hindrance and minimise heat loss. For the dry sand preparation, the sand was oven-dried at a temperature of 105 $^\circ$C for 24 h to eliminate any residual moisture content, ensuring it could be treated as a two-phase medium comprising solid particles and air, entirely free of water. After drying, the sand was allowed to cool to ambient temperature in a sealed container to prevent moisture absorption from the surrounding environment. Once cooled, the prepared sand was placed into the testing box for further experimental procedures. The sand was placed inside the tank in a layered manner (10 cm each) to ensure proper compaction of each layer. Also, at the same time maintaining the respective positions of the thermocouples. For compaction, a wooden tamping bar was used and an overall density of 1.65 g cm$^{-3}$ has been achieved to avoid settlement during saturation. To implement the saturation condition, a bottom-to-top approach was used. Four flexible rubber pipes at each corner were used, which run to the container’s bottom. In this process, the sand is saturated from bottom to top, thereby removing the trapped air from the intergranular pores. Commercially available water sealant was used to avoid any leakage from the container. After the container was filled up to the top, it was covered with thin, flexible aluminium sheets to avoid heat decampment. A drainage outlet was also provided.

### Thermocouples and heater

**Table 3:** Distance and position of the thermocouples.

Position of the thermocouples	Name and respective $\hbox {distances}^\textrm{a}$
Heater surface	A24, A10, A30, A31
Outside container	A0 (86), A8 (40)
Above heater	A7 (5), A11 (10), A14 (15), A26 (20), A9 (25), A27 (30)
Below heater	A20 (5), A4 (10), A2 (15), A1 (20), A3 (25), A19 (30), A15 (40), A12 (50), A25 (70)
Horizontal direction	A18 (5), A21 (10), A17 (15), A5 (20), A16 (25), A6 (30), A28 (40), A22 (50), A23 (55), A13 (85)

^a^Distances are in cm from the surface of the heater rod, with the distance mentioned in parentheses

The UGPS was simulated with a heater rod systematically devised with an electrical heater inside it. The heater rod (80 cm long, external diameter 5 cm) was placed firmly inside the container between two upright stands, and fixed between two supports (5cm wide each). The heating power was accurately measured using an inline power meter to ensure precise monitoring of energy input during the experiments. It was placed along the short edge (100 cm wide side), with its centre at a distance of 75 cm from the bottom of the container. The schematic diagram of the heater rod is shown in Fig. 2b. The temperatures and moisture data are recorded with National Instruments thermocouples and 15 cm long three-pin TDRs. 32 K type thermocouples (TCs) along with 8 Time-Domain Reflectometer (TDR). TDRs were set up at definite locations inside the box with distances measured from the surface of the heater rod (see Fig. 2b). The TCs had an operating temperature range of $-55$ $^\circ \textrm{C}$ to 550 $^\circ \textrm{C}$. The accuracy of the heating system is provided by the manufacturer with $\pm 0.5$ K and is cross-validated before installation. The K-type thermocouples bought from National Instruments showed a deviation smaller than $\pm 0.3$ K when tested against the resistance temperature detectors (RTDs) Pt-100 sensors. Furthermore, a separate test to evaluate the sensors was conducted with water filled in the box also fitted with 4 Pt-100 sensors. All thermocouples and the RTDs in water inside the box were within a range of 0.25 K, which is well within the permissible error range. Ambient temperatures were measured using two TC’s placed outside of the box. Four thermocouples are placed on the heater’s circumference, and the aluminium foil is wrapped to give uniform heat distribution from the cable. The average value of these four sensors is plotted as heater temperature in both static and dynamic thermal loading scenarios. Table 3 enlists the respective distances of the attached TCs. The heat source temperature of 90 $^\circ$C was selected to represent the maximum permissible conductor temperature for XLPE cables, as specified in international and U.S. standards, such as IEC 60840, IEC 62067, AEIC CS9, and ICEA S-108-720^39–42^. This temperature aligns with the maximum operating temperature defined for XLPE cables under normal conditions^39,41^. While the temperature at the outer cable sheath is typically lower due to the thermal resistivity of surrounding materials, using 90 $^\circ$C in this study provides a conservative approach to simulate worst-case scenarios. Additionally, this approach ensures the experimental setup reflects conditions, where heat dissipation and soil behavior are critical for cable performance under peak loading,^39,40^.

## Results and discussion

**Figure 3:**
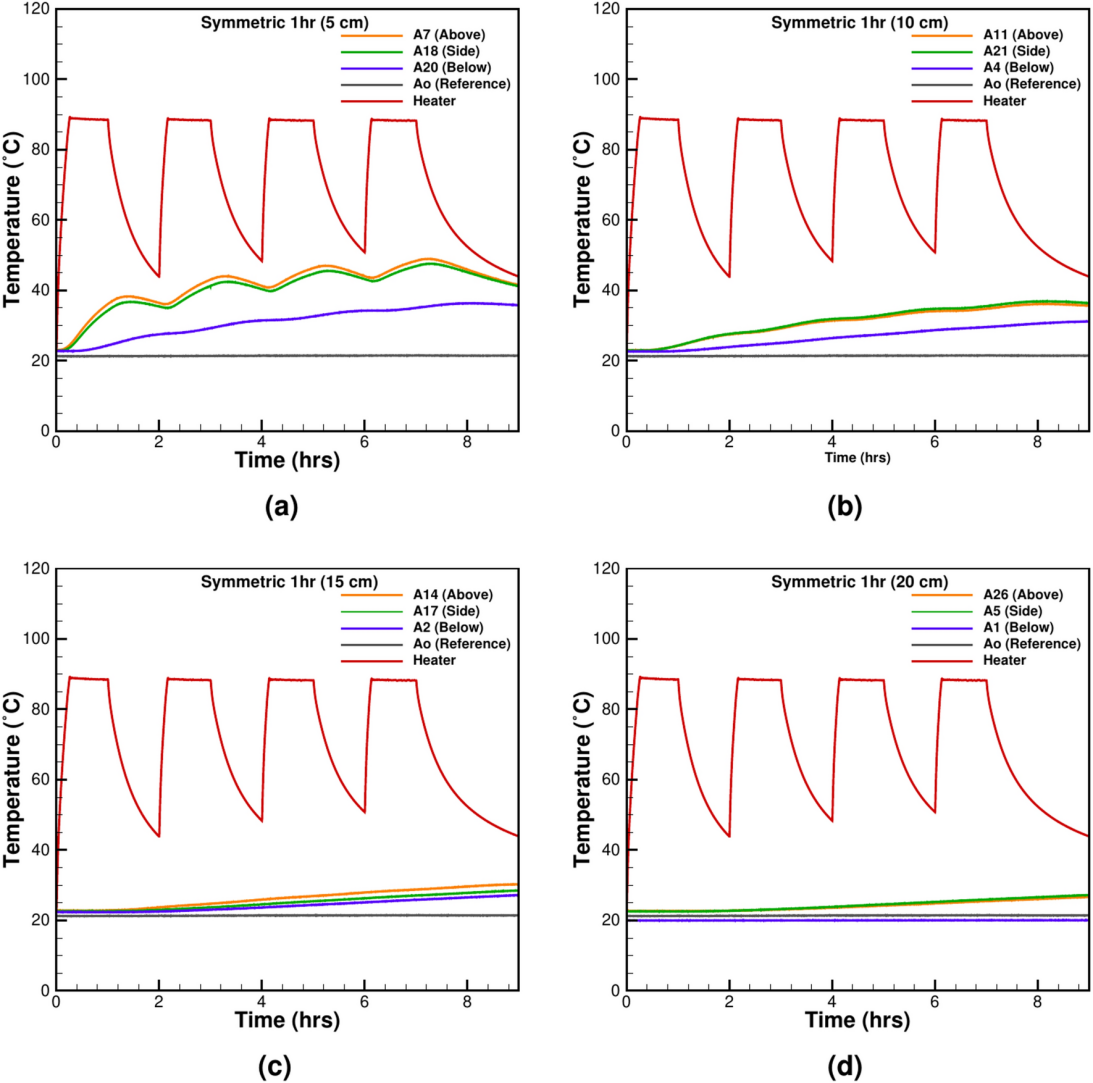
The symmetrical heating-cooling cycle of 1 h at various distances from the heater surface: (**a**) 5 cm, (**b**) 10 cm, (**c**) 15 cm, and (**d**) 20 cm.

**Figure 4:**
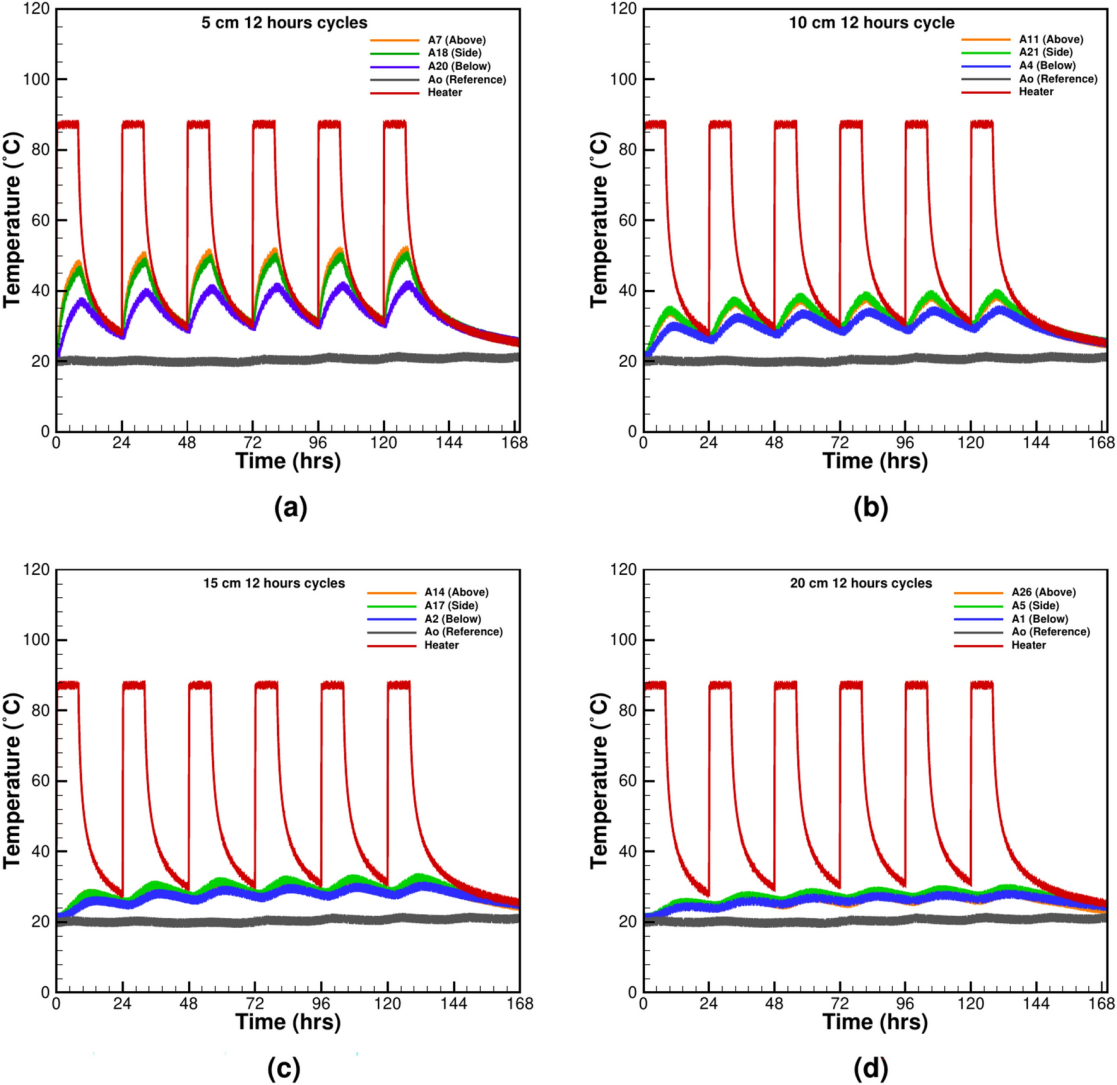
The symmetrical heating-cooling cycle of 12 h at various distances from the heater surface: (**a**) 5 cm, (**b**) 10 cm, (**c**) 15 cm, and (**d**) 20 cm.

The thermal performance of insulation materials under dynamic and static thermal conditions reveals significant insights into how environmental factors, particularly seasonal variations, influence insulation behavior. Seasonal changes, such as fluctuations in ambient temperature and soil moisture content, play a critical role in heat transfer mechanisms. For instance, during summer months, elevated temperatures and prolonged heat exposure contribute to increased heat accumulation, which can reduce insulation efficiency. Conversely, winter conditions slow down heat dissipation, influencing the thermal relaxation time and potentially increasing thermal stress. Furthermore, seasonal variations in soil moisture, as demonstrated in the static thermal loading tests with dry and saturated sand, highlight how moisture content affects thermal conductivity. Saturated conditions facilitate faster heat dissipation, while dry conditions result in localized heat buildup. The dynamic thermal loading test with only dry sand and the static thermal loading test with both dry and saturated sand is performed. The dynamic loadings are performed with three separate loadings of the equispaced heating-cooling, the shorter heating and longer cooling and the thermal relaxation time. The static thermal loading tests with dry and saturated sand are done to assess the long-term behaviour of the UGPS. The results are arranged in two separate subsections: the dynamic thermal loading with dry sand and the static thermal loading with dry and saturated sand.

### The dynamic thermal loading with dry sand

The thermal cyclic (symmetrical and unsymmetrical heating) and relaxation time tests are performed with dry sand with a maximum heater temperature of 70$^{\circ }C$. All the tests are performed at a surrounding temperature close to 20 $^{\circ }C$.

#### Symmetrical thermal heating-cooling cycles

Symmetrical thermal cycles are critical for understanding uniform heat dissipation in controlled environments. These cycles mimic idealized conditions often used in laboratory settings to test material stability and predict thermal responses. Cyclic thermal loading plays a crucial role in underground power cable systems, influencing thermal stress distribution and material durability. Repeated heating and cooling cycles impact soil properties and moisture migration, affecting the long-term performance and safety of the cables. Understanding these effects is essential for optimizing cable lifespan and ensuring efficient energy transmission in underground environments. Two tests are performed with equal heating and cooling cycles of 1 h and 12 h, as shown in Figs. 3 and 4. For both tests, it is observed that the heater’s operational effect is felt at all the TCs instantaneously. The 1-h test also shows that the cooling time is insufficient to bring the system to the original state (Fig. 3a–d), causing a gradual increase in the temperature of sand throughout the box. The effect of heating is felt at all the observation points ranging between 5 and 20 cm from the heater’s outer surface. The maximum influence is noticed at the TCs above the heater at 5 cm distance where the temperature reaches a value of 48.92 $^\circ {C}$. The minimum temperature of 26.69 $^\circ {C}$ is recorded below the heater. The observation suggests a permanent thermal charging of the system and is not desirable for the UGPS. The excess temperature reduces the thermal gradient between the heater and its surrounding and thus promotes heat storage and drying. For 12 h of heating-cooling cycles, the thermal charging effect is less significant. However, the intensity of charging is different from the 1-h cycles. After the first cycle for TC at 5 cm above the heater, a maximum temperature of 47.73 $^\circ \textrm{C}$ is recorded, which increases to 51.65 $^\circ \textrm{C}$ after five heating cycles. A similar trend is observed at the furthest TC at 20 cm from the heater, but the charging effect is pacified (Fig. 4d). In both experiments, it is evident that symmetrical heating-cooling cycles do not offer sufficient time for the surrounding sand to dissipate the extra heat given off by the heater. Therefore, the unsymmetrical heating-cooling experimental tests are performed to observe improvement in cooling of heated sand.

**Figure 5:**
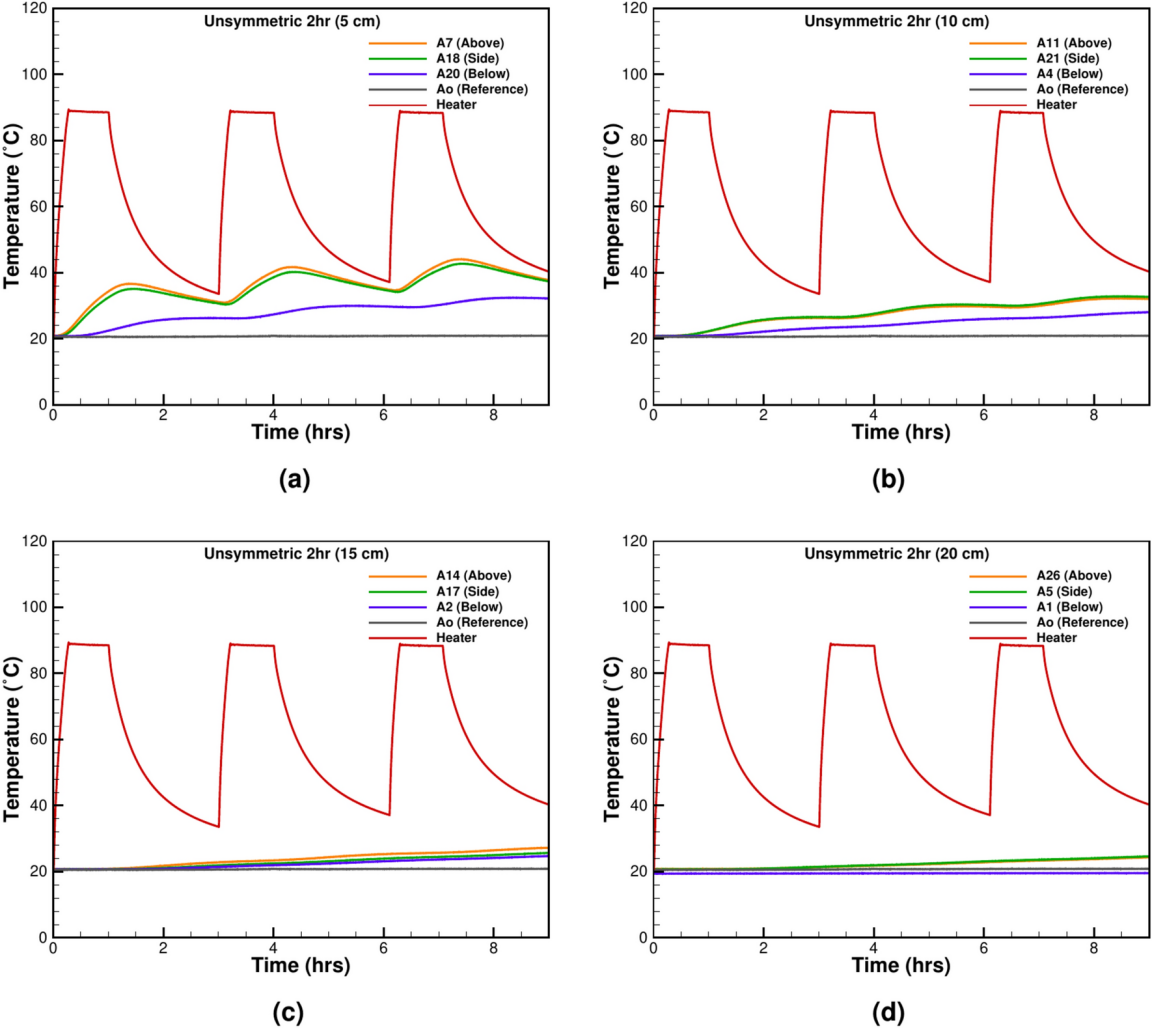
The unsymmetrical cycle with 1 h of heating and 2 h of cooling at various distances from the heater surface: (**a**) 5 cm, (**b**) 10 cm, (**c**) 15 cm, and (**d**) 20 cm.

#### Unsymmetrical thermal heating-cooling cycles

Unlike symmetrical cycles, unsymmetrical thermal heating-cooling cycles better represent real-world scenarios, such as localized heat sources in underground power cables or uneven thermal loads in building materials. The unsymmetrical loading tests are done with two thermal loading scenarios. The first test consists of three cycles of 1 h of heating and 2 h of cooling. The results show thermal charging taking place as well, but the intensity is lower than in the symmetrical case as more relaxation time is available for the system to dissipate heat into the surrounding (Fig. 5 a–d). A general trend could be identified for the surrounding temperature behaviour near the heat source as a nonlinear fluctuating rise (Fig. 5a, b) for the TCs at 5 and 10 cm from the heater’s surface. However, the rise is linear for the far-field TCs, as observed in (Fig. 5c, d) at 15 and 20 cm distance from the heater surface.

**Figure 6:**
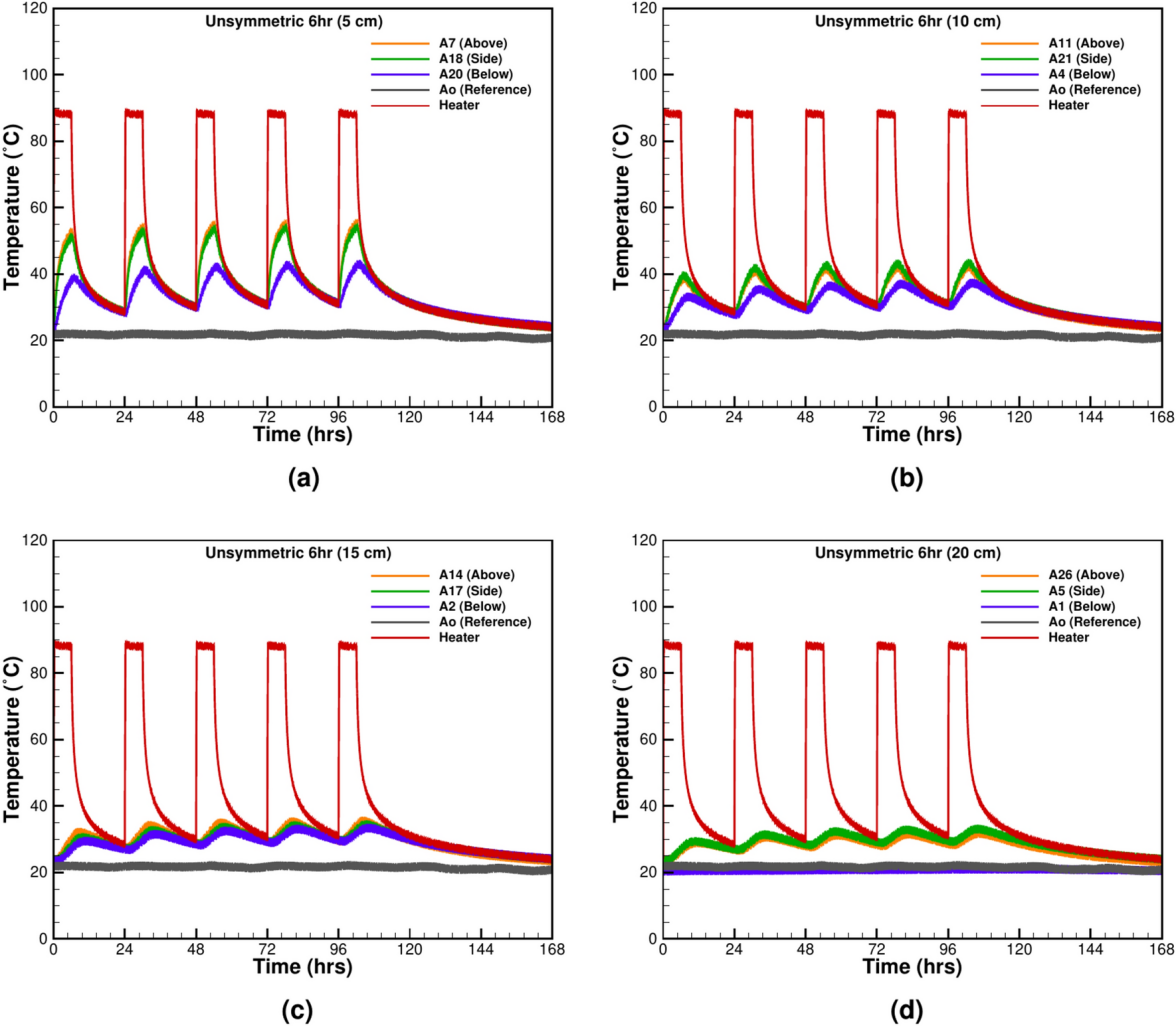
The unsymmetrical heating of a 6-h heating and 18-h cooling cycle at various distances from the heater surface: (**a**) 5 cm, (**b**) 10 cm, (**c**) 15 cm, and (**d**) 20 cm.

The second test is done with five cycles of 6 hours of heating and 18 h of cooling (Fig. 6a–d). The results suggest that the TCs at 5cm distance from the heater surface reach a maximum value of 36.61 $^\circ \textrm{C}$ in the first cycle and 44.05 $^\circ \textrm{C}$ in the last cycle. The change is significant and the system fails to reach a static equilibrium with the surrounding sand. Compared to 12 h of symmetric heating-cooling, a less sudden temperature rise is observed in Fig. 6d at the TC 20 cm away from the heater surface. The general trend is nonlinear and is similar to the near-field TCs recording. All the TCs show a nonlinear behaviour contrast to shorter heating-cooling cycles in Fig. 5d, where the general trend for far-field TCs was observed as linear. The dissimilarity arises due to the amount of energy given in the cases of 1 hour and 12 h of heating. Both results from Figs. 5 and 6 show that the cooling time with unsymmetrical heating is also insufficient to prevent thermal charging of the sand. Therefore, two separate tests are performed with a longer relaxation time, and results are given in the following section.

**Figure 7:**
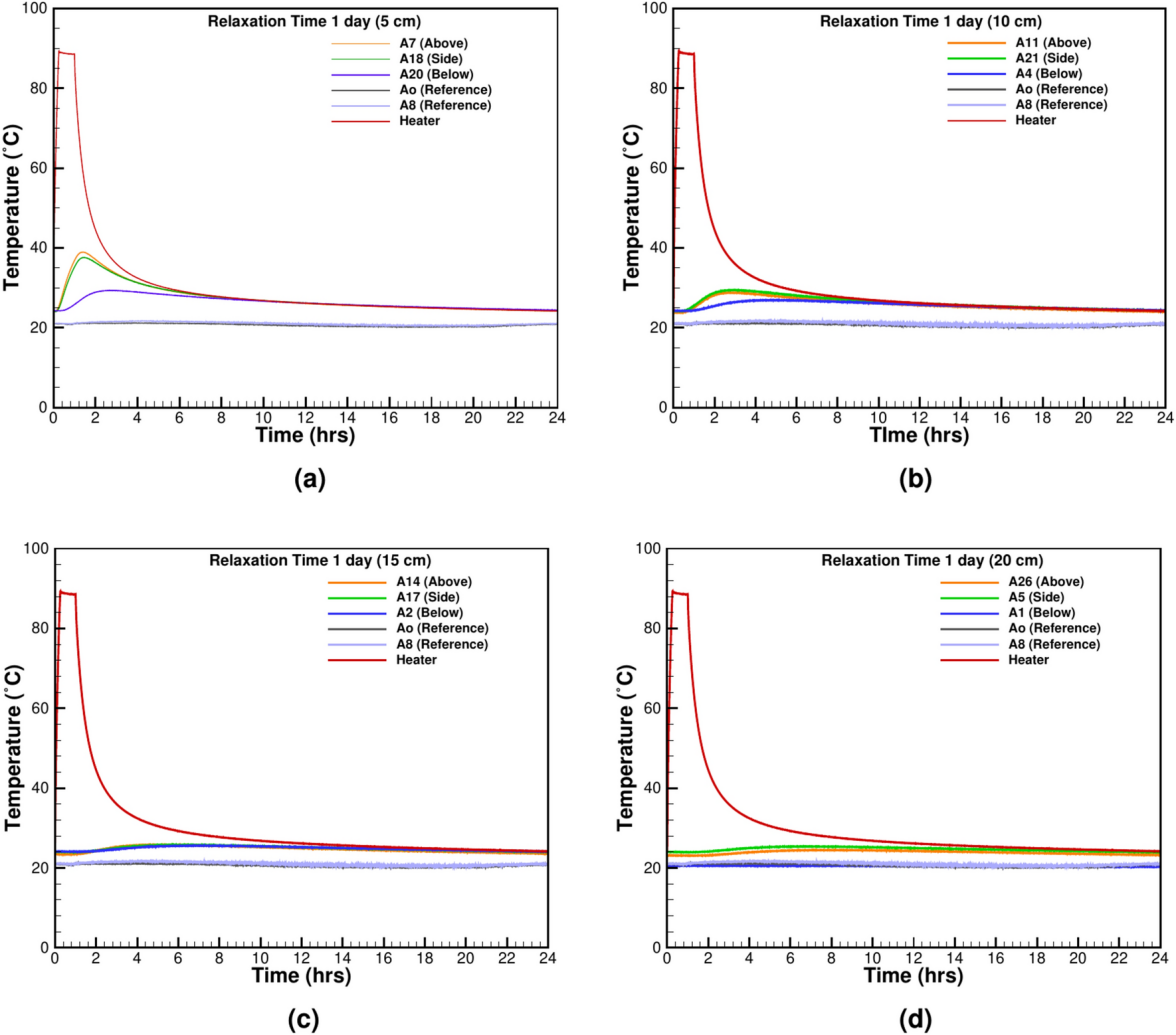
The thermal relaxation period after 1 h of heating followed by 23 h of cooling at various distances from the heater surface: (**a**) 5 cm, (**b**) 10 cm, (**c**) 15 cm, and (**d**) 20 cm.

**Figure 8:**
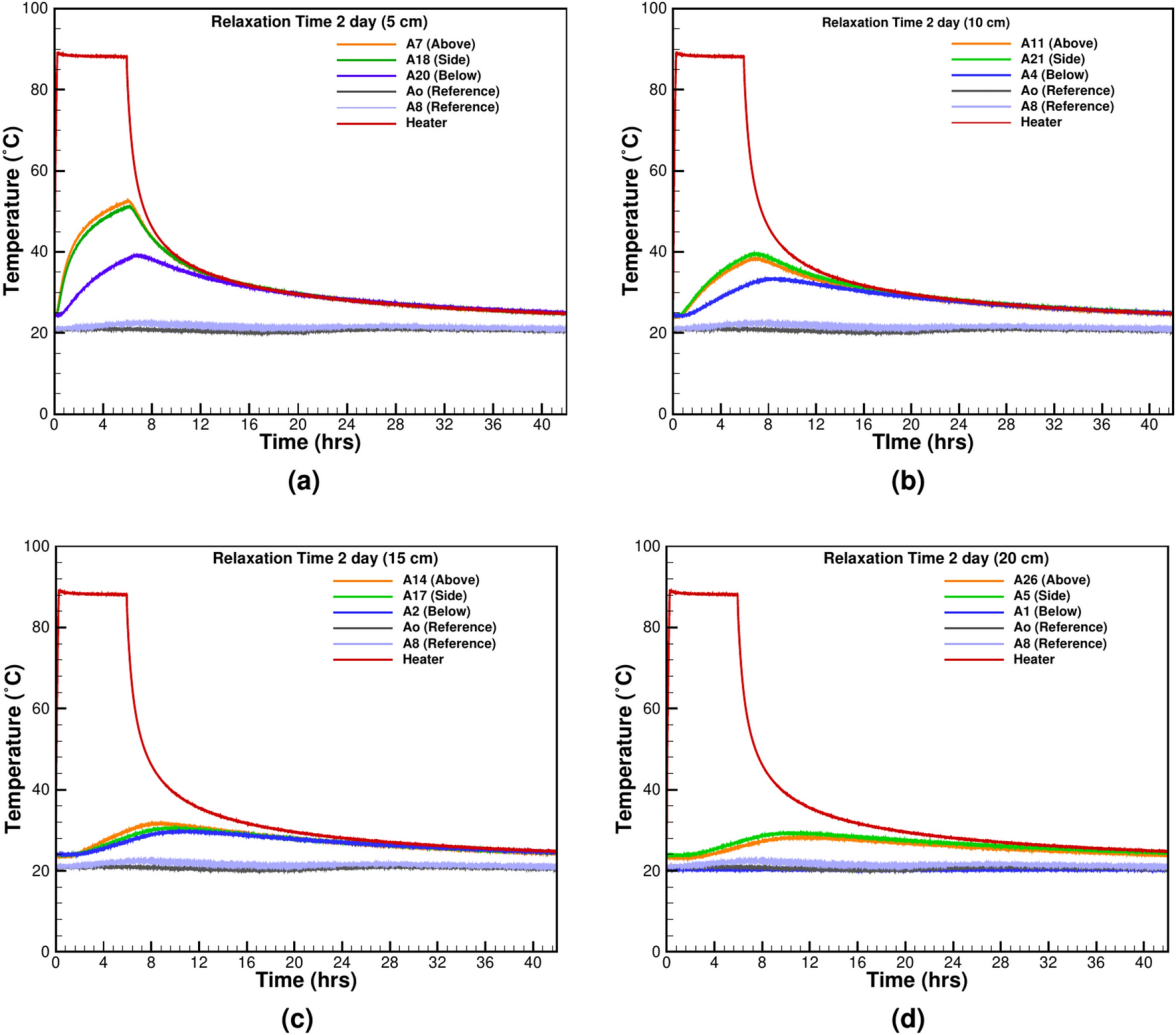
The thermal relaxation time after 6 h of heating followed by 36 h of cooling at various distances from the heater surface: (**a**) 5 cm, (**b**) 10 cm, (**c**) 15 cm, and (**d**) 20 cm.

#### Thermal relaxation time

Thermal relaxation time measures how quickly insulation returns to ambient conditions after thermal loading. It is a critical factor for assessing the material’s ability to recover from thermal stress. To properly quantify the relaxation time to prevent thermal charging, two tests with 1 and 6 h of heating and subsequent 23 and 36 h of cooling are done (Figs. 7 and 8). The near-field TCs at 5 cm and 10 cm distances experience the switching off of the heater at once (Fig. 7a, b), but the far-field temperature keeps on rising from the heat given by the heater during operation (Fig. 7c, d). After 8 h, the near-field TC as shown in Fig. 7a, b reaches equilibrium with the heater temperature, but the far-field TCs, 15 cm and 20 cm from the heater’s surface require 12 and 14 h, respectively. The relaxation time of 23 h is sufficient to bring the system close to the initial test temperature. The test with 6 h of heating and subsequent 36 hours of cooling is shown in Fig. 8a–d. The longer heating time provides more energy and prolongs the relaxation time. The near-field TCs respond immediately with the heater switching off (Fig. 8a, b), but the far-field TCs continue recording the rising temperature as shown in Fig. 8d. At all the measurement points, the heater’s temperature and the surrounding sand come to an equilibrium in an asymptotic fashion.

### Static thermal loading test with dry and saturated sand

**Figure 9:**
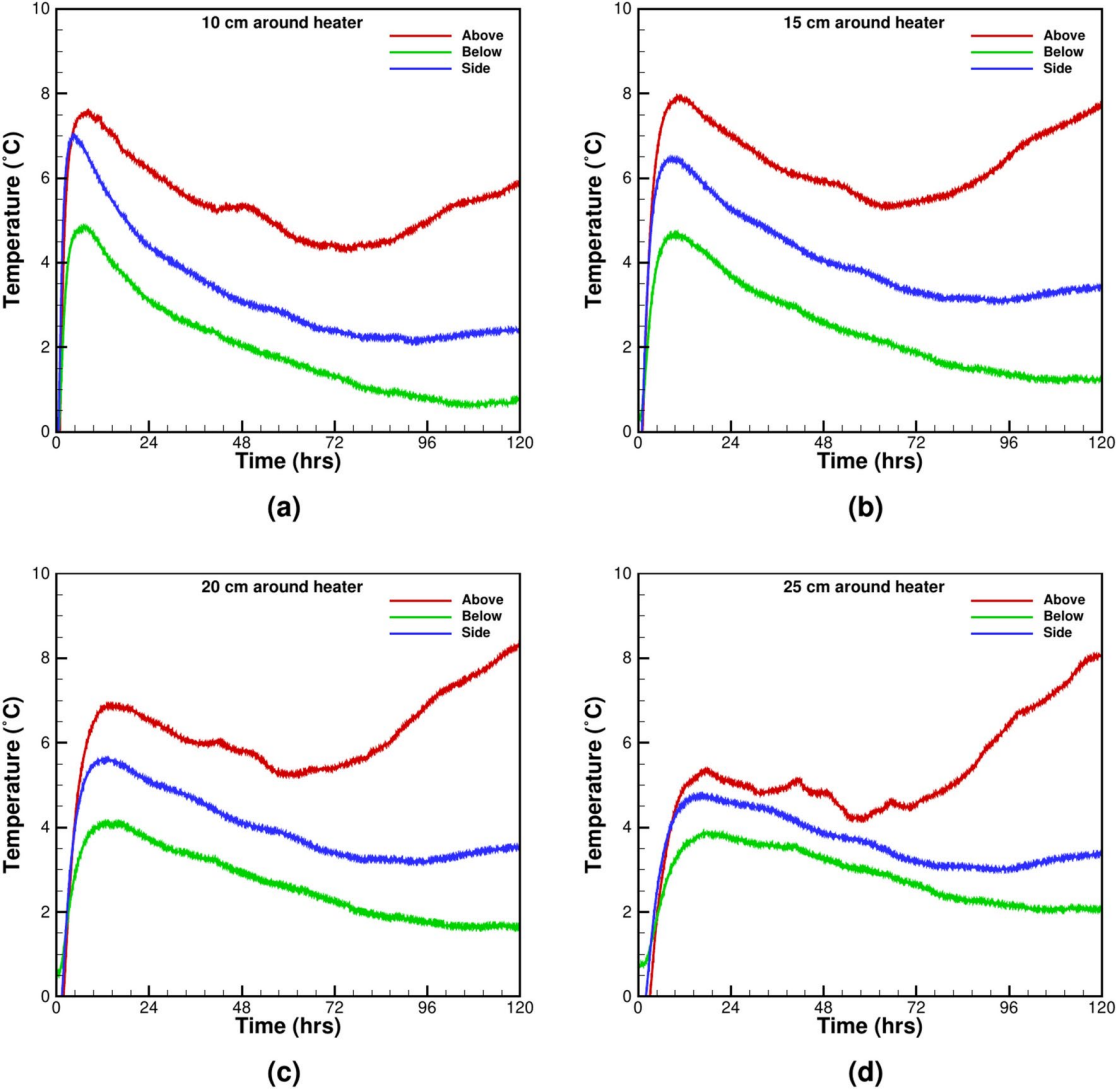
Temperature differences around the heater between the dry and saturated soil conditions at various distances from the heater surface: (**a**) 10 cm, (**b**) 15 cm, (**c**) 20 cm, and (**d**) 25 cm.

Static thermal loading tests assess the impact of soil moisture content on insulation performance. Moisture plays a critical role in determining thermal conductivity and heat dissipation, directly affecting insulation stability. Two identical tests with dry and saturated sand are performed under similar loading boundary conditions. A comparison of the temperature difference between dry and fully saturated sand is plotted for four profiles around the heater ranging between 10 and 25 cm. The thermal buoyancy effect is observed at all measuring locations with all the four TCs above the heater, as shown in Fig. 9a–d. The TCs are at equal distances from the heater’s surface show different behavior and indicate the non-uniform heat transfer characteristics. The temperature difference graph shows a sizeable initial difference and subsequent drop in the difference value. The initial rise is due to higher thermal conductivity of saturated sand, which facilitates heat transfer. Soon after, a partial thermal equilibrium with the surrounding is established and the difference between dry and saturated condition reaches an asymptotic constant value (Fig. 9a–d). The saturated sand shows a sharp rise in temperature above the heater after 72 h of heating and is visible at all thermocouples above the heater (Fig. 9a–d). The sudden increase of the temperature above the heater arises with the formation of convection cells. No significant rise is observed in the horizontal plane and at the TCs below the heater. The convection heat transfer becomes dominant above the heater with increasing distance (Fig. 9a–d). The results indicate a density-driven buoyancy flow of liquid water and uneven channelized heating of the surrounding soil. Although channelized heating helps remove heat from the UGPS, it is undesirable under crop fields and causes damage to the plants’ roots. The thermal buoyancy effect due to convective cells’ formation has been previously reported for fully saturated porous media^43^. It is shown that below a critical Reynolds number, convective cell flow is two-dimensional and time-independent but becomes time-dependent three-dimensional flow above the threshold, which depends inversely on heater length and burial depth. The UGPS burial depth is optimized for trench construction costs, ranging from 1.5 to 2.0 m to avoid damage from agricultural plugs and surface digging. In the dimension-reduced laboratory-scaled model, it is fixed at 45 cm. The heater position is not varied, resulting in a two-dimensional density-driven flow around it.

**Figure 10:**
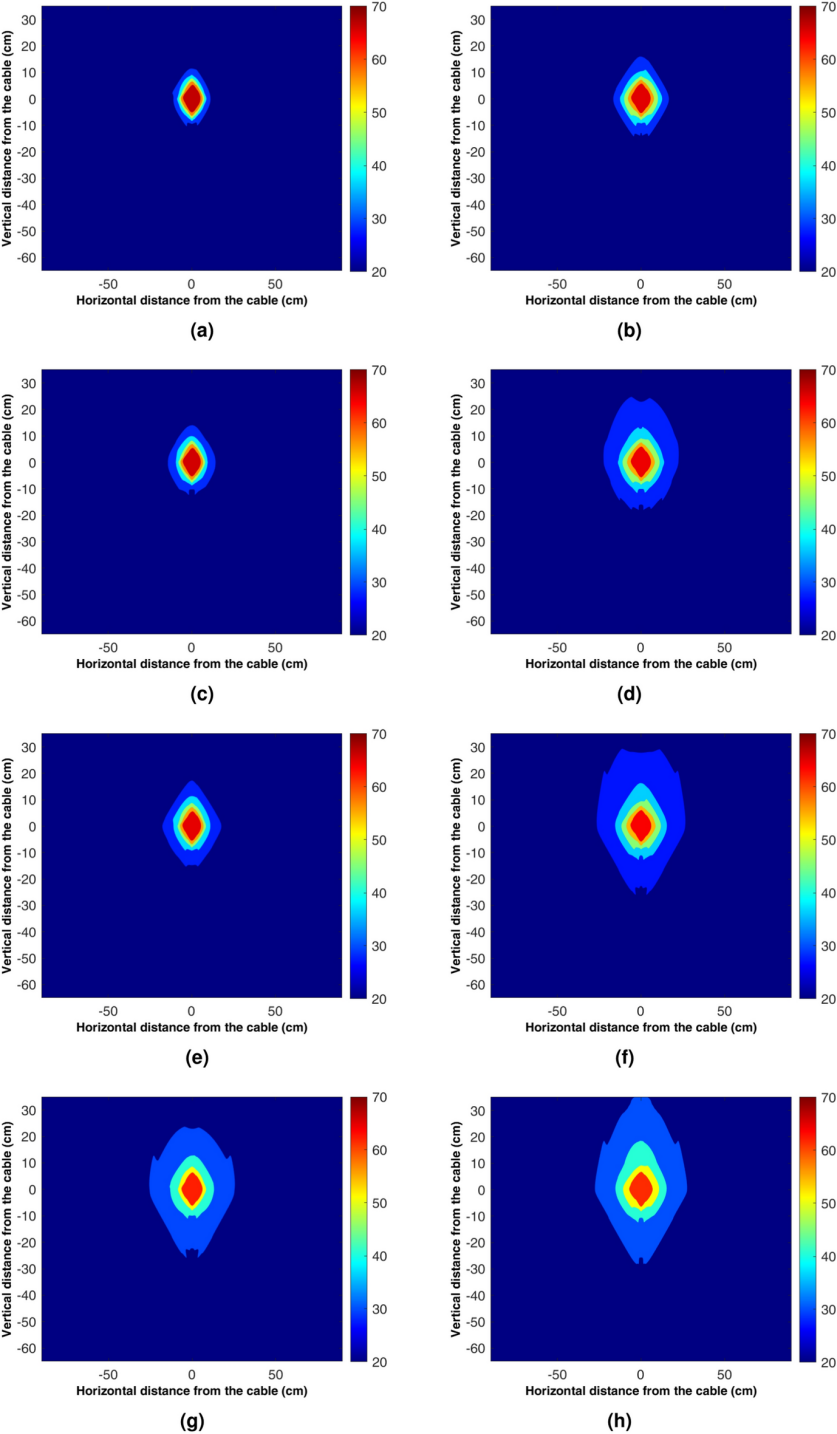
Experimental contour plots for the evolution of temperature in the box under dry and saturated conditions after 3, 6, 12, and 120 h. The left-side figures correspond to dry conditions, while the right-side figures represent fully saturated sand.

Figure 10 shows the 2D temperature contour plot generated from the measured data for dry and fully saturated conditions. As measurements are performed only in half of the box, the symmetry condition is considered for full-field plotting. The cubic interpolation function is used to smooth the figure between measurement points. The contour plots offer better insight into the evolution of the convection cells for saturated sand around the heater with time. Figure 10a, c, e, g and b, d, f, h show the evolution of temperature in the dry and saturated conditions, respectively, after 3, 6, 9, and 120 hours of heating. The temperature near the heater reaches 70 $^\circ {C}$ and is shown with the color bar. The temperature field in all dry conditions is restricted in the vicinity of the heater and gradually increases as time passes. For all the saturated sand tests, the temperature fields are broader and encompass a more significant area than the dry sand. Also, in Fig. 10. h, which shows the temperature field after 120 h, the thermal buoyancy is visible with a candle flame-like shape above the heater. The numerical and theoretical models for temperature field calculation around the UGPS do not consider the buoyancy effect, thus resulting in incorrect temperature distribution and heat loss computation. The channelized heat loss from the UGPS below a paddy crop field, at a crossing with the gas or water pipelines in high water table regions and near water bodies should be considered for correct temperature field calculation.

## Conclusion

This study experimentally investigates the thermal behavior of dry and saturated sand surrounding a heated cylinder, designed to simulate an underground power cable system under both cyclic and static thermal loading. In dry sand, cyclic thermal loading induces thermal charging, progressively reducing the sand’s capacity to dissipate heat efficiently. Thermal relaxation experiments, performed with heating durations of 1 and 6 h, demonstrate an asymptotic cooling trend as time progresses. This means that after the heat source is turned off, the sand’s temperature decreases rapidly at first but gradually slows down, approaching a stable equilibrium temperature over time. These findings highlight the critical role of soil moisture in thermal management for energy systems. Saturated sand enhances heat transfer through buoyancy-driven convection after 72 h, improving cooling efficiency for underground cables and geothermal systems. However, localized heat concentration above the heater in saturated conditions necessitates careful design to prevent hot spots. Distinct heat transfer mechanisms in dry and saturated soils provide valuable insights for optimizing thermal systems, ensuring efficiency, safety, and reliability in geotechnical and energy applications. The findings of this study have direct applications in optimizing underground power cable design and improving thermal management strategies for energy infrastructure. Insights from this research can aid in developing efficient soil backfill materials, enhancing cooling mechanisms, and mitigating thermal stress in high-voltage systems. These applications are particularly relevant for sustainable energy networks, where effective heat dissipation is crucial for reliability and efficiency. The study reveals the pivotal role of natural convection in governing temperature dynamics and enhancing heat dissipation in underground power cable systems. This insight is particularly vital for regions with high water tables, proximity to water bodies, agricultural zones, and areas prioritizing sustainable energy solutions. By integrating these principles into cable system design, engineers can optimize thermal performance across diverse soil conditions, fostering robust, efficient, and sustainable energy infrastructure that meets the demands of a rapidly evolving world.

## Data Availability

The datasets generated and/or analyzed during the current study are available from the corresponding author upon reasonable request.

## List of symbols

a: Volumetric air content
*c*, $$C_p$$: Specific heat $$(\textrm{J} \, \text {kg}^{-1} \, \text {K}^{-1})$$
C: Volumetric heat capacity $(\mathrm{J \, m}^{-3}\, \textrm{K}^{-1})$
D: Diffusion coefficient of water vapour in air $(\textrm{m}^{2}\, \textrm{s}^{-1})$
$\hbox {D}_{T}$: Macroscopic diffusivity for moisture transport due to $\nabla T (\textrm{m}^{2}\, \textrm{s}^{-1}\, \textrm{K}^{-1})$
$\hbox {D}_{\theta }$: Macroscopic diffusivity for moisture transport due to $\nabla \theta _{1} (\textrm{m}^{2}\, \textrm{s}^{-1})$
e: Empirical factor
g: Acceleration due to gravity $(\mathrm{m\,s}^{-2})$
h: Relative humidity
H: Specific enthalpy $$(\mathrm{J \, kg}^{-1})$$
${\textbf {k}}$: Unit vector in the z-direction
K: Hydraulic conductivity $$(\mathrm{m\,s}^{-1})$$
L: Latent heat of evaporation $(\mathrm{J\,kg}^{-1})$
M: Molar mass $$(\mathrm{kg \, mol}^{-1})$$
$\hbox {p}_{v}$: Partial pressure of water vapour (Pa)
P: Total gas pressure (Pa)
q: Flux density for mass $(\mathrm{kg \, m}^{-2}\, \textrm{s}^{-1})$
$\hbox {q}_{h}$: Flux density for heat $(\mathrm{J \,m}^{-2}\, \textrm{s}^{-1})$
R: Universal gas constant $(\mathrm{J\, mol}^{-1} \, \textrm{K}^{-1})$
S: Porosity
t: Time (s)
T: Temperature (K)
x,y: Horizontal coordinate (m)
z: Vertical coordinate, positive downward (m)

## Greek symbols

$\varepsilon$: Low value of $\theta _{l}$
$$\zeta$$: $$(\nabla T)_{a}/ \nabla T$$
$\theta$: Volumetric moisture content
$\lambda$: Effective thermal conductivity $(\mathrm {W \, m}^{-1} \, \textrm{K}^{-1}$)
$$\psi$$: Moisture potential $$(\mathrm m$$)
$$\rho$$: Density $$( \mathrm{kg\, m}^{-3})$$

## Subscripts

0: Reference value
a: Air
d: Drying
h: Heat
k: Critical
l: Liquid
m: Moisture
r: Reversal
s: Saturation
v: Vapour
w: Wetting
